# Patterns of Relative Bacterial Richness and Community Composition in Seawater and Marine Sediment Are Robust for Both Operational Taxonomic Units and Amplicon Sequence Variants

**DOI:** 10.3389/fmicb.2022.796758

**Published:** 2022-02-07

**Authors:** Zak Kerrigan, Steven D’Hondt

**Affiliations:** Graduate School of Oceanography, University of Rhode Island, Narragansett, RI, United States

**Keywords:** operational taxonomic unit, amplicon sequence variant, marine sediment, marine water column, tag sequencing, bacteria

## Abstract

To understand the relative influences of operational taxonomic units (OTUs) and amplicon sequence variants (ASVs) on patterns of marine microbial diversity and community composition, we examined bacterial diversity and community composition of seawater from 12 sites in the North Atlantic Ocean and Canadian Arctic and sediment from two sites in the North Atlantic. For the seawater analyses, we included samples from three to six zones in the water column of each site. For the sediment analyses, we included over 20 sediment horizons at each of two sites. For all samples, we amplified the V4–V5 hypervariable region of the 16S ribosomal RNA (rRNA) gene. We analyzed each sample in two different ways: (i) by clustering its reads into 97%-similar OTUs and (ii) by assigning sequences to unique ASVs. OTU richness is much higher than ASV richness for every sample, but both OTUs and ASVs exhibit similar vertical patterns of relative diversity in both the water column and the sediment. Bacterial richness is highest just below the photic zone in the water column and at the seafloor in the sediment. For both OTUs and ASVs, richness estimates depend on the number of sequences analyzed. Both methods yield broadly similar community compositions for each sample at the taxonomic levels of phyla to families. While the two methods yield different richness values, broad-scale patterns of relative richness and community composition are similar with both methods.

## Introduction

In recent years, high-throughput sequencing (HTS) has dominated studies of microbial diversity and community composition in marine environments (Inagaki et al., 2006; Bienhold et al., 2016; Kirkpatrick et al., 2019) and other environments (He et al., 2015; Tremblay et al., 2015; Berry et al., 2017; Glassman and Martiny, 2018). The ability to sequence either a large number of samples on a single sequencing run or deep sequencing of a small number of samples (millions to tens of millions of amplicons per sample) generates terabytes of data for a single study. For this purpose, some studies have relied on operational taxonomic units (OTUs; Huse et al., 2010; Jing et al., 2013; Bienhold et al., 2016; Sheik et al., 2018; Kirkpatrick et al., 2019; Pohlner et al., 2019), whereas others have relied on amplicon sequence variants (ASVs; Callahan et al., 2017; Hoshino et al., 2020; Fadeev et al., 2021). Studies that rely on OTUs typically cluster sequences at an operationally defined level of similarity (usually 97%; Ward et al., 1998; Roesch et al., 2007; Huse et al., 2010; He et al., 2015; Mysara et al., 2017). This clustering is intended to reduce the impact on diversity estimates of errors that accumulate during PCR amplification and genetic sequencing (Huse et al., 2010; Kunin et al., 2010). Alternatively, studies that rely on ASVs use algorithms intended to remove the errors associated with sequencing and return individual, unique sequences that represent individual taxa (Rosen et al., 2012; Eren et al., 2013; Callahan et al., 2016; Edgar, 2016). These sequences (ASVs or ESVs—exact sequence variants) differ from each other by as little as a single nucleotide change (Rosen et al., 2012; Callahan et al., 2016; Edgar, 2016).

Reliance of previous marine studies solely on OTUs or on ASVs has hindered synthesis of OTU-based studies with ASV-based studies to draw broad conclusions about distributions of microbial richness and community composition in the open ocean and marine sediment. Fortunately, patterns have begun to emerge from recent studies that compared the two methods using mock communities (Nearing et al., 2018; Prodan et al., 2020) and/or communities from diverse non-marine environments (soil: Nearing et al., 2018; Joos et al., 2020, plant-associated communities: Joos et al., 2020, fecal matter: Nearing et al., 2018; Prodan et al., 2020, and dairy products: Xue et al., 2018). These previous studies have mostly focused on the differences between the OTU and ASV (we include ESVs, zero-radius OTUs, and other exact sequence nomenclature) methods, and the shortcomings of each. Here, we present an investigation that highlights the similarities and dissimilarities of OTU-based results and ASV-based results for the same microbial communities from seawater and marine sediment.

Our goals are to (i) assess the extent to which interpretations of marine microbial diversity and community composition depend on the analytical approach chosen (OTU or ASV) and (ii) identify how their respective results can be used to make conclusions regardless of each pipeline. For this purpose, we have characterized the bacterial diversity and community composition of seawater and marine sediment by clustering genetic sequences into 97%-similar OTUs with the mothur pipeline (Schloss et al., 2009) and identifying ASVs from the same samples with the DADA2 pipeline (Callahan et al., 2016).

## Materials and Methods

### Sample Collection and DNA Extraction

**Figure 1:**
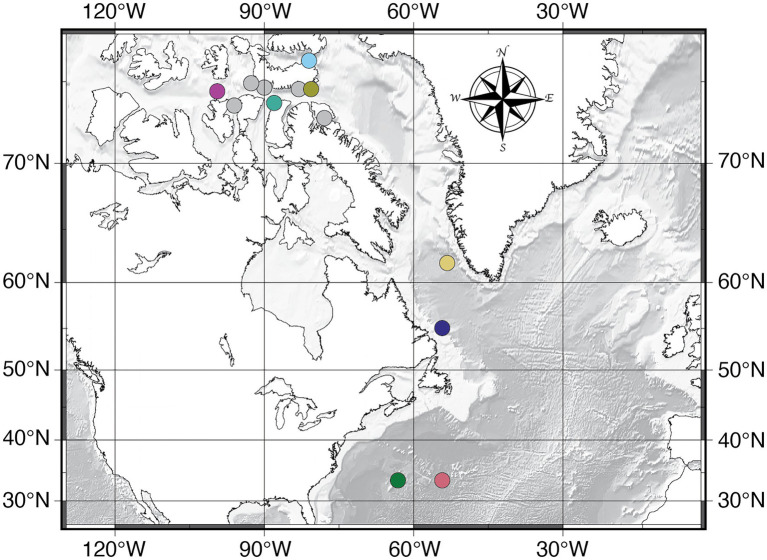
Sampling locations for this study. Data presented in this manuscript includes all sample locations except for the data in Figures 2, 5, which only include the colored circles. The colors correspond to the same colors in Figures 2, 5.

We collected the seawater samples from 12 geographic locations in the western North Atlantic (*RV Falkor* expedition FK003b and *RV Knorr* expedition KN223) and Canadian Arctic (*Icebreaker Oden* expedition NWP2019; Figure 1). We collected the water and measured oceanographic properties using a 24-Niskin bottle CTD rosette (SeaBird SBE 9). Once the CTD/Niskin system was on deck, we used a 50 in, D Bar clamp to seal the top and bottom of each 12 L Niskin bottle. The vent plugs of each Niskin bottle were replaced with t-fittings, and a chain of plastic tubing was attached to each t-fitting to connect all the bottles to a common manifold which was connected to an air compressor. A cylindrical, 0.22 μm Sterivex membrane filter (Millipore Sigma, Billerica, MA, United States) was connected to the petcock valve of each Niskin bottle, and the pressure manifold was pressurized between 8 and 10 psi to create a filtration rate between 66 and 80 ml/min to filter the entire contents of each bottle. Once each Niskin bottle was empty, we removed and sealed the filters and stored them in the shipboard −80°C freezers until they were transported to the University of Rhode Island (URI), Graduate School of Oceanography (GSO) for post-expedition DNA extraction using the DNeasy PowerWater Sterivex Kit (Qiagen, Germantown, MD, United States) following the manufacturer’s protocol. We targeted a combination of standard depths and oceanographic horizons (sea surface, O_2_ minimum, chlorophyll maximum, thermocline, and deep-water) and analyzed two to six sampling depths at each station between 3 and 5,500 m water depth depending on location (Supplementary Table S1).

We collected the sediment samples from two locations in the North Atlantic (*RV Knorr* expedition KN223, Sites 15 and 16; Figure 1). We collected sediment samples from multiple sediment depths in cores that collectively span the interval from the seafloor to as much as 40 meters below seafloor (mbsf; Supplementary Table S2). Sediment coring was conducted using a multi-coring sampler [0–30 cm below seafloor (cmbsf)], a gravity-driven coring device (up to 4 mbsf), and a longer, piston-coring device (up to 40 mbsf). We subsampled the sediment using sterile 60 cc syringes that had been modified by removing the tapered tip and immediately froze the subsamples at −80°C for post-expedition DNA extraction at URI, GSO. For each sediment sample, we extracted DNA in duplicate, 0.25 g subsamples using the DNeasy PowerSoil Pro Kit (Qiagen, Germantown, MD, United States) following the manufacturer’s protocol, and pooled the duplicate extracts for amplification. Sediment age was approximated for each depth by dividing basement age (Müller et al., 2008) by sediment thickness (Divins, 2003), and assuming a constant sediment deposition rate. Both sites exhibit similar dissolved chemistry profiles, with oxygen undetectable below 2–3 cmbsf (Murray and Party, 2014).

### PCR Amplicon Construction and Sequencing

From each extract, we amplified the V4–V5 hypervariable region of the 16S rRNA gene using forward and reverse primers from Parada et al. (2016). We performed a 20-μl PCR reaction for each sample which contained a mixture of 0.4 μl Platinum SuperFi II DNA Polymerase (Invitrogen, Carlsbad, CA, United States), 4 μl SuperFi II Buffer (5×), 0.2 mM dNTPs, 0.5 μM of each primer, and 0.1 μl bovine serum albumin (Thermo Scientific, Carlsbad, CA, United States). We amplified each water sample in a single PCR reaction with 5 μl of DNA template and each sediment sample in triplicate with 12 μl of DNA template. To account for possible PCR reagent contamination during PCR amplification, we amplified three samples (one for each batch of polymerase used) containing only laboratory water and no extract. Additionally, we completed the full DNeasy PowerWater Sterivex protocol using a sterile Sterivex filter for each kit and the full DNeasy PowerSoil Pro protocol with no sediment for each kit to account for possible kit contamination. The thermal cycler program for all reactions began with an initial denaturation temperature of 98°C for 30 s followed by 35 cycles of 98°C for 10 s, 60°C for 10 s, and 72°C for 15 s, followed by a final extension of 72°C for 5 min. The triplicate sediment samples were then pooled, and we cleaned all of the samples using the Agencourt AMPure PCR Purification Kit (Beckman Coulter Life Sciences, Indianapolis, IN, United States). Finally, all samples were sent to the University of Rhode Island Genomics and Sequencing Center and sequenced on an Illumina MiSeq using the Illumina MiSeq V3 chemistry at 2 × 300 cycles (NCBI BioProject PRJNA718862).

### Sequence Analyses

To process and analyze the sequences as 97%-similar OTUs, we used mothur v.1.42.3 (Schloss et al., 2009) and followed the mothur MiSeq standard operating procedure (SOP; Kozich et al., 2013, revision 6/24/19).^1^ To process and analyze the same sequences as ASVs, we used DADA2 v.1.16.0 (Callahan et al., 2016) and followed the DADA2 Pipeline Tutorial (1.16).^2^ In both cases, we removed from all samples any reads that were sequenced from the PowerWater and PowerSoil extraction kits, as well as any reads from the PCR negative controls (described above), to account for possible lab and reagent contamination. Due to the low number of sequences in each of these negative controls, this removal had no impact to the results of each pipeline.

For our OTU analysis, we randomly subsampled all samples to 10,000 sequences prior to clustering to make direct comparisons when conducting community analysis (Weiss et al., 2017). We chose this value for the following reasons: (i) rarefaction of samples to the same number of sequences provides a more accurate comparison when analyzing clustered, genetic data (Weiss et al., 2017; Cameron et al., 2021; Ramakodi, 2021), (ii) when comparing broad-scale patterns of relative taxonomic abundance, our previous study showed that deep-sea marine sediment and open ocean water column samples with 10,000 sequences provide the same results as the non-rarefied samples with much greater numbers of sequences (Kerrigan et al., 2019), and (iii) 10,000 sequences per sample is considered sufficient to capture most diversity data (Cameron et al., 2021) and well above the value that may begin to insert errors during analysis (Cameron et al., 2021; Chiarello et al., 2021). Unless otherwise stated, we performed an opti-clust (Westcott and Schloss, 2017) clustering analysis at 97% sequence similarity. Additionally, we removed singletons and doubletons of OTUs from each sample to mitigate the likelihood of including OTUs based solely on sequencing error. To investigate the effect of sequence number on estimates of taxonomic richness using OTUs, we compared the OTU richness based on total reads to the OTU richness of the same samples based on 10,000 randomly subsampled sequences. All samples exhibited similar effects, and we have chosen two of the North Atlantic deep-sea sediment samples as representatives to illustrate the consequences and implications of the random subsampling.

For our standard ASV analysis, we completed the DADA2 pipeline with all sequences. We made this choice because DADA2 does not incorporate rarefaction into its standard pipeline, and our goal was to compare the “standard” methods an environmental researcher might use while conducting a genetic study. However, to additionally investigate the effect of sequence number on estimates of taxonomic richness using ASVs, we compared the non-rarefied ASV richness for each sample to the ASV richness of the same sample randomly subsampled to 10,000 reads. As with the OTU results, this subsampling consistently changes the ASV-based results. We illustrate that change with the same two North Atlantic sediment samples that we used to illustrate the effect of random subsampling on OTU-based results. Unless otherwise stated, the ASV results presented are based on the non-rarefied samples. By default, the DADA2 algorithm removes singletons as part of its process.

### Ecological Metrics and Statistical Analyses

We used R v 4.0.4 (R Core Team, 2021) to perform all diversity, community composition, and statistical analyses, primarily employing packages *phyloseq* (McMurdie and Holmes, 2013), *ggplot2* (Wickham, 2016), *dbplyr* (Wickham et al., 2021), and *vegan* (Oksanen et al., 2020). Our diversity analyses included calculations of OTU richness, as well as the Shannon and Simpson diversity metrics. We also calculated Shannon’s Equitability Index (*E_H_* = *^H^/ln S*; Pielou, 1966), where *H* is the Shannon Index and *S* is the taxonomic richness for each sample. For each diversity metric, we directly compared the values between the OTU and ASV analysis and plotted the linear regression with resulting Pearson’s *r* and value of *p*. We also calculated the mean of the OTU and ASV values for all samples, and the associated SE. Finally, we calculated and plotted abundance curves for each sample with both the maximum number of sequences in each sample and the 10 k-sequence subsampled data for both OTUs and ASVs.

Our community composition analyses included both taxonomic assignment of OTUs and ASVs from phylum to family and dissimilarity calculations at the OTU/ASV level. For taxonomic classification, we assigned phylogenies to each OTU/ASV based on the Silva reference database (v138) and then constructed relative taxonomic composition profiles for each sample by calculating the contribution of each taxon as a percentage of the whole sample. To compare the OTU/ASV-level community composition between samples, we calculated two-dimensional Bray–Curtis distance matrices and plotted the results using Nonmetric Multidimensional Scaling (NMDS) ordination. We also calculated an environmental fit ordination and overlaid the resulting parameter vectors (salinity, temperature, fluorescence, and oxygen) on the NMDS figures to help explain and visualize underlying trends in sample groupings.

## Results

### Taxonomic Diversity

**Figure 2:**
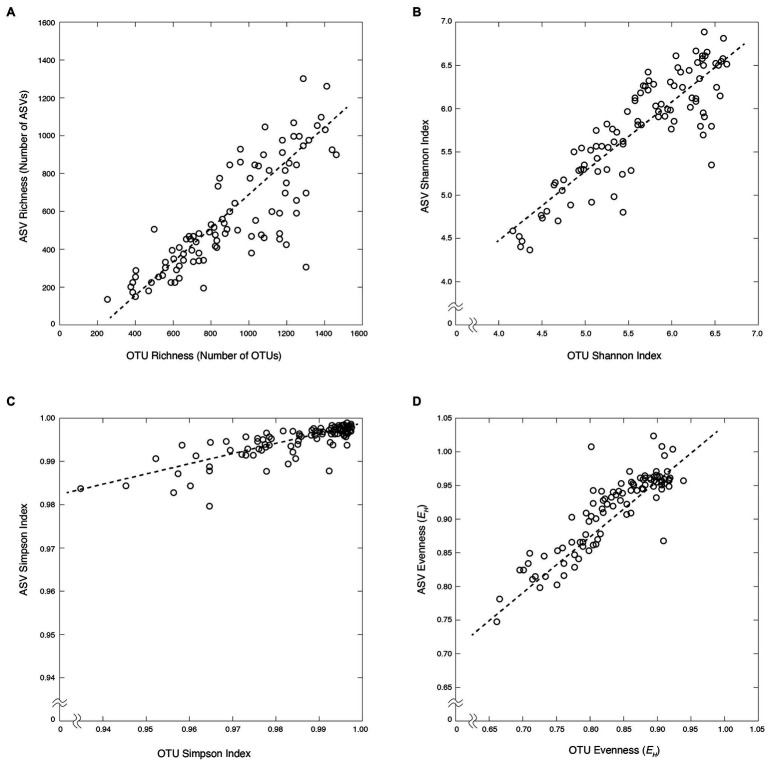
Bacterial richness profiles of the water column and marine sediment analyzed with both OTU- and ASV-based methods. All Arctic sample sites exhibit similar profiles, and only four of the nine Arctic sites are presented here for ease of visualization. Sample colors correspond to sample sites plotted in Figure 1. **(A)** Bacterial richness of water column OTUs (right) and ASVs (left). **(B)** Bacterial richness of marine sediment OTUs (right) and ASVs (left).

**Figure 3:**
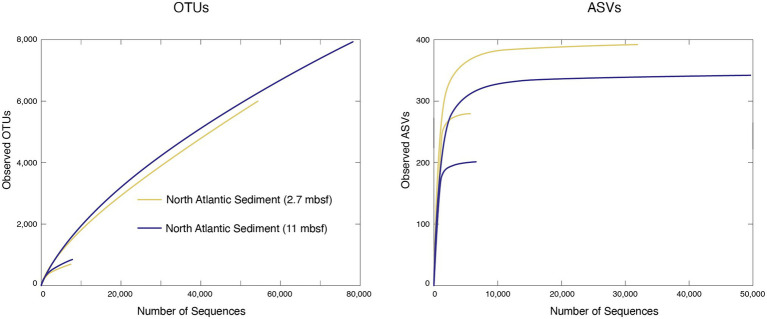
Bacterial diversity metrics of OTUs vs. ASVs for the water column (blue) and marine sediment (red). **(A)** Bacterial richness (*r* = 0.82, *slope* = 0.76). **(B)** Shannon index (*r* = 0.87, *slope* = 0.80). **(C)** Simpson index (*r* = 0.80, *slope* = 0.24). **(D)** Bacterial evenness (*r* = 0.88, *slope* = 0.76).

In total, 30,651 bacterial OTUs were generated from all samples by clustering a total of 564,364 sequences with the mothur pipeline (Supplementary Tables S3 and S4). Eighteen thousand six hundred ninety-six bacterial ASVs were generated from all samples from a total of 2,402,375 sequences with the DADA2 pipeline (Supplementary Tables S3 and S4). The large difference in total initial sequences for the two pipelines was due to rarefication of samples prior to clustering in the mothur pipeline and not in the DADA2 pipeline. Although more than four times as many sequences were used to determine total ASVs over OTUs, nearly twice as many OTUs were generated as ASVs [mean = 1,102 ± 36 (SE) and 744 ± 38 (SE) for OTUs and ASVs, respectively]. Despite this difference in their total numbers, OTU and ASV profiles exhibit similar vertical patterns of relative taxonomic richness (total number of OTUs or ASVs in each sample) in both the water column and the sediment (Figure 2). In the water column, bacterial richness is highest at or just below the chlorophyll-a maximum (1,474 OTUs and 1,264 ASVs). In the sediment, bacterial richness is highest at the sediment–water interface (925 OTUs and 733 ASVs) and diminishes with increasing sediment depth and age, consistent with previously established trends (Walsh et al., 2016; Kerrigan et al., 2019; Kirkpatrick et al., 2019). The relative richness of bacterial OTUs in each sample is linearly correlated to the relative richness of bacterial ASVs (*r* = 0.82, value of *p* < 0.00001), with average ASV richness generally lower than OTU richness (*slope* = 0.76; Figure 3).

The Shannon and Simpson diversity indices for OTUs and ASVs are similarly correlated [*r* = 0.87 (value of *p* < 0.00001) and *r* = 0.80 (value of *p* < 0.00001), respectively]. Shannon diversity values are relatively high for both OTUs and ASVs (4.16–6.90) with ASV-based values generally higher than OTU-based values (*slope* = 0.80). The average ASV-based Simpson diversity values are also high and span a narrower range (0.96–0.99) than the OTU-based values (0.93–0.99; *slope* = 0.24; Figure 3).

Community evenness, based on Shannon’s Equitability Index (*E_H_*), was also similar between OTU and ASV data. This index is a measure of community evenness on a scale from 0 to 1, where 1 is a value of total evenness (in which each OTU or ASV in the sample contains the same number of sequences). As with taxonomic richness and the diversity indices, OTU-based *E_H_* values are linearly correlated to ASV-based *E_H_* values (*r* = 0.88, value of *p* < 0.00001; Figure 3). Also, as with the diversity indices, ASV-based *E_H_* values are generally higher and span a narrower range that OTU-based *E_H_* values (Figure 3).

**Figure 4:**
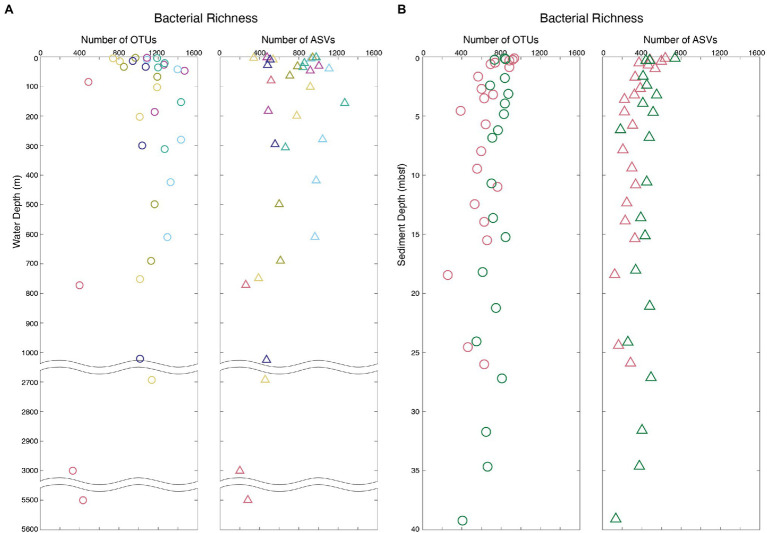
Bacterial taxonomic abundance curves of operational taxonomic units (OTUs; left) and amplicon sequence variants (ASVs; right) for two marine sediment samples at both their full number of sequences, and a subsampled, 10,000-sequence data set.

To illustrate the effect of sequence numbers on estimates of taxonomic richness for both OTUs and ASVs, we analyzed all of the samples at both their entirety of amplicon sequences and randomly subsampled to 10,000 sequences per sample, as described above. Every sample exhibits similar issues with regard to subsampling. Consequently, we have chosen two of the North Atlantic deep-sea sediment samples as representatives to illustrate the effects and implications of the random subsampling (Figure 4). Before subsampling and processing with either pipeline these samples (1 and 2) contained approximately 67 and 100 k sequences, respectively. After completing the mothur OTU clustering of the full data set, sample 1 contained 56,630 sequences with 5,975 OTUs and sample 2 contained 79,914 sequences with 7,981 OTUs. At the end of the DADA2 ASV pipeline with the full data set, sample 1 contained 30,924 sequences with 391 ASVs, and sample 2 contained 49,501 sequences with 343 ASVs. The OTUs and ASVs yield very different rarefaction curves. For both samples, the OTU curve continues with a positive slope, whereas the ASV curve approaches an asymptote very quickly. Reducing the total number of reads by random subsampling significantly changes the rarefaction curves for both OTUs and ASVs. After randomly subsampling both samples to 10,000 sequences, the OTU pipeline retained 7,426 sequences with 598 OTUs in sample 1 and 7,478 sequences with 758 OTUs in sample 2. The ASV pipeline retained 6,631 sequences with 278 ASVs and 6,350 sequences with 200 ASVs for the same samples. For both OTUs and ASVs, the subsampled OTU and ASV numbers yield rarefaction curves with much lower rates of increase than the curves defined by the full numbers of sequences in each sample (Figure 4). The ASV-based curves for the subsamples approach much lower asymptotes than the asymptotes for the ASV-based curves for the total sequences in each sample.

### Community Composition

**Figure 5:**
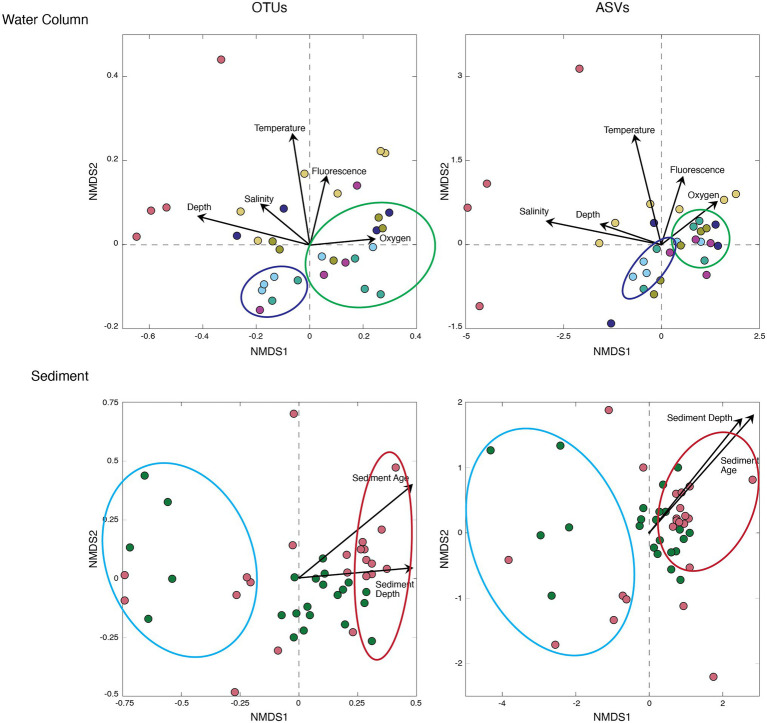
Bray–Curtis Nonmetric Multidimensional Scaling (NMDS) of the water column (top) and marine sediment (bottom). All Arctic samples exhibit similar groupings, and only four of the nine Arctic sites are presented here for ease of visualization. Sample colors correspond to sample sites plotted in Figure 1. OTUs (left) and ASVs (right) are overlayed with environmental factors for both the water column (salinity, temperature, fluorescence, and oxygen) and sediment (sediment depth and sediment age).

To compare the OTU-based community composition to the ASV-based community composition for all samples, we calculated Bray–Curtis distance matrices and plotted the results using NMDS ordination (Figure 5). To help explain and visualize underlying trends in sample groupings, we added environmental factors to the NMDS figures (salinity, temperature, fluorescence, and oxygen for the water column, and sediment depth and sediment age for the sediment). For both the water column samples and the sediment samples, the OTU-based results and the ASV-based results grouped the samples similarly, with each grouping explained by environmental factors. For the water column, both the OTU-based analysis (*NMDS stress* < 0.12) and the ASV-based analysis (*NMDS stress* < 0.10) grouped the shallow water and Arctic water samples to the right, which corresponded to higher oxygen and fluorescence values, and grouped the deeper and more southerly samples to the left, which corresponded to higher salinity and temperature values (Zinger et al., 2011). For the sediment, the OTU-based results (*NMDS stress* < 0.18) and the ASV-based results (*NMDS stress* < 0.14) both grouped the deeper (older) samples to the right along the *x*-axis (Figure 5).

**Figure 6:**
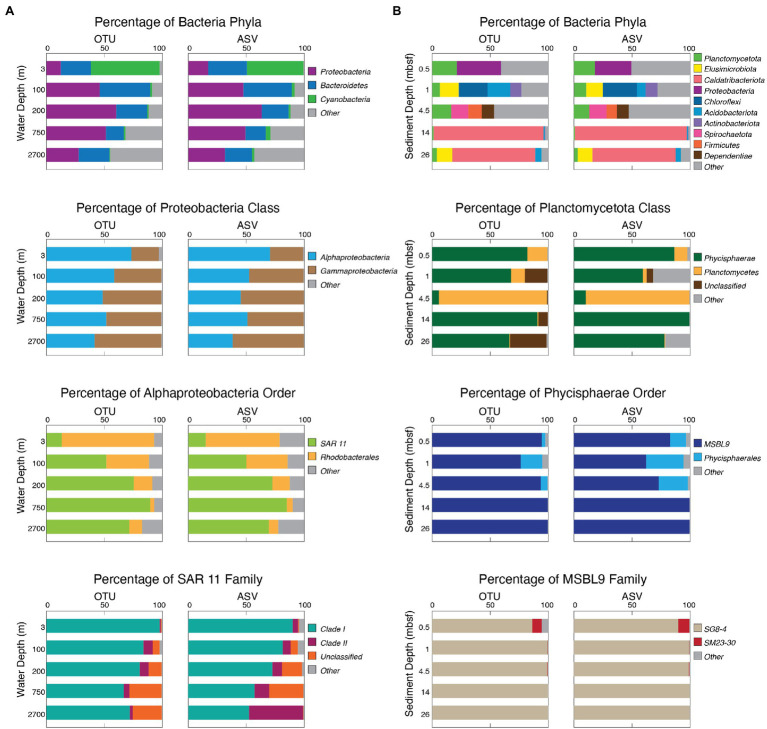
Bacterial community composition as analyzed with both OTUs and ASVs in the water column and marine sediment. **(A)** Water column community composition of the Bacteria domain, Proteobacteria phylum, Alphaproteobacteria class, and SAR 11 order with OTUs (left) and ASVs (right). **(B)** Marine sediment community composition of the Bacteria domain, Planctomycetota phylum, Phycisphaerae class, and MSBL9 order with OTUs (left) and ASVs (right).

In addition to OTU- and ASV-level community similarity, we mapped each OTU and ASV in our study to the Silva reference database (v138). We then calculated the relative abundance of each OTU and each ASV in each sample and compared the bacterial results of each sample at the phylum, class, order, and family level. To illustrate the similarities, we chose five of the most diverse samples from one water column location and five of the most diverse samples from one sediment location and compared the OTU- and ASV-based results of the most prevalent taxa at each phylogenetic level (Figure 6). These examples show that the OTU-based results and the ASV-based results yield very similar patterns of taxonomic abundance. With either OTUs or ASVs, the water column populations are dominated by *Proteobacteria* at the phylum level, *Alphaproteobacteria* at the class level, and *SAR 11* at the order level. OTUs and ASVs also yield similar abundance patterns for the sediment samples, where community composition changes with sediment age and depth. For both OTUs and ASVs, shallow samples are more evenly distributed across varying phyla, and the deeper samples are dominated by the phylum *Caldatribacteriota*. However, each sample for both methods contain the phylum *Planctomycetota*, and most of these are dominated by the class *Phycisphaerae* and the order *MSBL9*.

## Discussion

### Taxonomic Richness

The vertical profiles of relative bacterial richness in both the water column and sediment were similar in nature between the OTU and ASV analysis (Figure 2). This result indicates that the variation in relative bacterial richness from one marine environment to another is captured by both OTUs and ASVs. In agreement with previous OTU-based studies, bacterial richness peaks in the water column at or just below the photic zone and decreases with increasing ocean depth (Signori et al., 2014; Walsh et al., 2016). Also, in agreement with previous OTU-based studies, bacterial richness in the sediment is highest at the seafloor and decreases with increasing depth (Walsh et al., 2016; Petro et al., 2017; Kirkpatrick et al., 2019).

While the OTU and ASV pipelines produced similar vertical patterns of relative richness, the large difference between OTU richness and ASV richness for each sample reinforces previous recognition that the choice of OTUs or ASVs significantly impacts estimates of total bacterial richness (Glassman and Martiny, 2018; Nearing et al., 2018; Xue et al., 2018; Joos et al., 2020; Prodan et al., 2020). In this study, the mothur-based OTU richness of each sample averages 1.77 times the DADA2-based ASV richness of that sample. This could be caused either by the OTU pipeline accumulating and clustering sequencing errors or by the ASV pipeline discarding real sequences during filtering and error modeling. However, the ratio of OTUs to ASVs can vary considerably by altering the user-defined parameters in each pipeline or by analysis with other pipelines (Prodan et al., 2020). In some cases, the ratio is even reversed; in comparing the UPARSE OTU clustering and ASV algorithms, Glassman and Martiny (2018) found bacterial OTU richness of leaf litter communities to be, on average, half the ASV richness. However, in this study of marine communities and in the previous studies of non-marine communities, patterns of relative variation in taxonomic richness between samples remain robust regardless of the method used. This result, of robust between-sample variation in relative richness within studies with large differences in richness between studies, does not solely depend on the choice of bioinformatic pipeline. It also depends on the specific 16S rRNA region amplified. For example, previous studies have shown that V4-based taxonomic richness closely matches the OTU richness based on the entire 16S rRNA gene (Tremblay et al., 2015), whereas V6-based richness is consistently higher (Liu et al., 2008; Youssef et al., 2009). Additionally, different hypervariable regions may show biases toward different bacterial families (Mysara et al., 2017). Despite these differences, different hypervariable regions yield similar patterns of relative richness and class-level community composition for marine communities (Kerrigan et al., 2019).

The absence of an asymptote in the OTU-based rarefaction curves for the full data sets in Figure 4 shows that these OTUs cannot be used to identify an absolute number of taxa in these samples. This issue is further demonstrated by the difference between the 10,000-read OTU-based rarefaction curves and the full data set OTU-based curves, which shows that the slope and rate of change in slope of these curves depend on the number of sequences used to define the curves. This issue is also known from studies of soil and plant-associated bacterial communities (e.g., Roesch et al., 2007; Joos et al., 2020). It has been ascribed to OTU instability, with membership in a particular OTU dependent on the number of clustered sequences (He et al., 2015), and to accumulation of sequencing errors with increasing numbers of reads (Joos et al., 2020). Unlike the OTU-based curves in Figure 4, the ASV-based curves for the same sample approach asymptotes quickly. However, as with the OTU-based curves, reducing the total number of reads to 10,000 per sample greatly changes the ASV-based rarefaction curves, leading to a much lower asymptote for both samples (Figure 4). As with the OTU-based rarefaction curves, this issue has been previously observed for ASV-based rarefaction curves for bacterial communities associated with soil and plants (Joos et al., 2020). For ASV-based curves, the dependence of slope and asymptote on the total number of reads used to define the curve may be at least partly due to removal of singletons by the standard ASV pipeline. Although both the OTU approach and the ASV approach appear to capture similar patterns of variation in relative richness from one sample to another, this dependence of rarefaction curves on total read numbers indicates that the outputs of the standard pipelines for OTUs and ASVs are not good predictors of absolute richness.

### Community Composition

Our NMDS analyses reveal similar ordination patterns for both OTU-based community composition and ASV-based composition in both the water column and the sediment (Figure 5). In the water column, samples associated with the chlorophyll-a maximum are grouped together across samples, and distinct from those from deeper depths (Figure 5). Each ordination also distinguishes the much deeper and more southerly water samples taken from the North Atlantic from the other samples (Figure 5). Inclusion of the environmental factors specific to each sample as an ordination overlay to the NMDS distribution shows that OTU-based ordination differences between OTUs and ASVs exhibit similar relationships to the environmental properties that control community composition. In short, as similarly seen in a recent study of leaf litter bacteria and fungi (Glassman and Martiny, 2018), the OTU and ASV pipelines produce similar cross-sample diversity patterns for bacterial communities in seawater and marine sediment.

Direct comparison shows that OTU-based taxonomic assignments broadly match ASV-based assignments for the communities in both the water column and the sediment (Figure 6). This similarity between the OTU- and ASV-based results is illustrated in Figure 6 by the water column communities from our site located off the Southeast coast of Greenland, where the five sampled horizons, representing five distinct oceanographic zones, were dominated by *Proteobacteria*, *Bacteroidetes*, and *Cyanobacteria* in similar proportions using both OTUs and ASVs. At each taxonomic level from phylum to family, we show the finer scale composition of the most abundant or most common taxon (Figure 6). Although there are some small differences, such as some of the ASV reads mapping to the *SAR 11 Clade II* while OTU reads remain *unclassified* in the deepest water sample, the results are generally very similar. Both pipelines returned results that broadly agree with previous OTU-based investigations of water column community composition in different regions of the open ocean (DeLong et al., 2006; Brown et al., 2009; Jing et al., 2013; Tseng et al., 2015; Medina-Silva et al., 2018).

To compare OTU-based composition to ASV-based composition for the marine sedimentary communities, Figure 6 shows results for five of the most diverse deep-sea sediment samples from the North Atlantic. Both the OTU and ASV pipelines reveal similar communities in each sample, from the seafloor to 26 meters below seafloor (mbsf; Figure 6). These samples range in sediment age from approximately 0 to approximately 7 million years (Ma; Figure 6). As with our example seawater communities, the major taxonomic contributors are the same for both the OTU-based results and the ASV-based results and agree with the broad community composition of previous studies (Inagaki et al., 2006; Nunoura et al., 2016; Petro et al., 2017; Kerrigan et al., 2019).

In short, the OTU pipeline and the ASV pipeline yield very similar patterns of relative richness and community composition for both seawater and marine sediment. In combination with recent comparisons of OTU-based results and ASV-based results for bacterial and fungal communities in leaf litter (Glassman and Martiny, 2018) and for bacterial and fungal communities associated with soil and plants (Joos et al., 2020), this result indicates that the OTU approach and the ASV approach yield similar ecological conclusions for diverse marine and terrestrial environments. This consistent similarity points to the potential of using both approaches simultaneously to check for consistency and gain confidence in the ecological conclusions from environmental genetic investigations.

## Conclusion

While many studies in recent years have highlighted the differences between bioinformatic pipelines, our comparison of OTU-based results and ASV-based results demonstrates that both methods yield similar patterns of relative taxonomic richness and evenness in seawater and marine sediment. Additionally, NMDS groupings of the sampled communities and their relationships to environmental factors are similar for both methods, and for individual samples, from the phylum level to the family level, community composition is broadly similar for both methods. Given this degree of similarity, simultaneous use of both OTU and ASV approaches may be useful for strengthening broad conclusions about diversity and community composition, and differences between the output of the two approaches may be useful for highlighting areas where more detailed scrutiny is required.

## Data Availability Statement

The data sets presented in this study can be found in online repositories. The names of the repository/repositories and accession number(s) can be found in the article/Supplementary Material.

## Author Contributions

ZK designed and executed the study with significant input from SD. ZK conducted all laboratory analyses and bioinformatics, with the exception of amplicon sequencing. ZK wrote the manuscript with significant input from SD. All authors contributed to the article and approved the submitted version.

## Funding

This study was funded by the Center for Dark Energy Biosphere Investigations (C-DEBI) (Grant NSF-OCE-0939564).

## Conflict of Interest

The authors declare that the research was conducted in the absence of any commercial or financial relationships that could be construed as a potential conflict of interest.

## Publisher’s Note

All claims expressed in this article are solely those of the authors and do not necessarily represent those of their affiliated organizations, or those of the publisher, the editors and the reviewers. Any product that may be evaluated in this article, or claim that may be made by its manufacturer, is not guaranteed or endorsed by the publisher.

## Footnotes

^1^ http://mothur.org/wiki/miseq_sop/

^2^ https://benjjneb.github.io/dada2/tutorial.html

## Supplementary Material

Supplementary Table S1 Water-column samples.

Supplementary Table S2 Sediment samples.
