# Supplementary material for: Patterns of Relative Bacterial Richness and Community Composition in Seawater and Marine Sediment Are Robust for Both Operational Taxonomic Units and Amplicon Sequence Variants
Source: Front Microbiol. 2022 Feb 7;13:796758. doi: 10.3389/fmicb.2022.796758 (PMC8859096; doi:10.3389/fmicb.2022.796758)
Supplement: Supplementary Table S1 — Water-column samples. [file Data_Sheet_1.pdf]

Cruise	*Site*	*Lat/Long*	*Water Depth (m)*	*Sample Depth (m)*
Northwest Passage Project	3	75°57'N	630	7
July - August 2019		080°50'W		40
				280
				420
				609
	5	72°47'N	780	20
		078°15'W		60
				380
				600
				770
	16	74°26'N	712	1
		081°34'W		30
				65
				500
				690
	21	74°40'N	146	10
		093°07'W		25
				136
	23	74°26'N	191	1.5
		099°17'W		26
				45
				182
	24	73°45'N	220	1
		096°49'W		30
				100
				210
	30	74°28'N	263	27
		090°31'W		251
	40	73°29'N	330	1.5
		088°55'W		20
				35
				150
				308
	51	74°29'N	450	1.8
		083°11'W		30
				225
				425
R/V Falkor Cruise 003b	8	61°53'N	2890	3
July 2012		053°16'W		10.4
				101
				201
				751
				2690
	10	55°09'N	1032	10.3
		054°16'W		29
				299
				1046
R/V Knorr Cruise 223	15	33°29'N	5510	81
October - December 2014		054°10'W		771
				3000
				5505
