# Supplementary material for: Patterns of Relative Bacterial Richness and Community Composition in Seawater and Marine Sediment Are Robust for Both Operational Taxonomic Units and Amplicon Sequence Variants
Source: Front Microbiol. 2022 Feb 7;13:796758. doi: 10.3389/fmicb.2022.796758 (PMC8859096; doi:10.3389/fmicb.2022.796758)
Supplement: Supplementary Table S2 — Sediment samples. [file Data_Sheet_2.pdf]

Cruise	*Site*	*Lat/Long*	*Water Depth (m)*	*Sample Depth (mbsf)*	*Sediment Age (Ma)*
R/V Knorr Cruise 223	15	33°29'N	5510	0.13	0.03
October - December 2014		054°10'W		0.26	0.07
				0.52	0.14
				0.65	0.17
				0.2	0.05
				0.9	0.24
				1.7	0.45
				3.2	0.85
				4.62	1.23
				2.7	0.72
				3.5	0.93
				5.7	1.52
				8	2.13
				9.5	2.53
				11	2.9
				12.5	3.33
				14	3.72
				15.5	4.12
				18.5	4.92
				21.5	5.72
				24.5	6.52
				26	6.92
	16	33°41'N	4545	0.13	0.01
		057°37'W		0.26	0.03
				0.39	0.04
				0.2	0.02
				1.7	0.17
				2.4	0.24
				3.9	0.39
				4.77	0.48
				0.2	0.02
				3.2	0.32
				4.7	0.47
				6.2	0.63
				6.9	0.7
				10.7	1.08
				13.7	1.38
				15.2	1.54
				18.2	1.84
				21.2	2.14
				24.2	2.44
				27.2	2.75
				31.7	3.2
				34.7	3.5
				39.2	3.96
