# Supplementary material for: Patterns of Relative Bacterial Richness and Community Composition in Seawater and Marine Sediment Are Robust for Both Operational Taxonomic Units and Amplicon Sequence Variants
Source: Front Microbiol. 2022 Feb 7;13:796758. doi: 10.3389/fmicb.2022.796758 (PMC8859096; doi:10.3389/fmicb.2022.796758)
Supplement: Supplementary file 3 [file Data_Sheet_3.pdf]

			Sequences			*Output*	
Cruise	*Site*	*Sample Depth (m)*	*Raw*	*Final*		*# of OTUs*	*# of ASVs*
				OTU	*ASV*		
Northwest Passage Project July - August 2019	3	7	63,148	10,000	34,100	1,097	909
		40	69,065	10,000	37,245	1,391	1,104
		280	78,318	10,000	32,357	1,413	1,032
		420	68,588	10,000	32,726	1,322	976
		609	63,936	10,000	33,732	1,294	958
	5	20	74,428	10,000	44,629	1,294	1,306
		60	65,726	10,000	38,297	1,273	1,005
		380	69,844	10,000	34,756	1,214	858
		600	84,054	10,000	39,338	1,240	1,070
		770	36,741	10,000	19,151	1,207	428
	16	1	73,177	10,000	46,016	957	938
		30	51,458	10,000	33,299	848	784
		65	41,104	10,000	25,728	1,197	703
		500	45,119	10,000	27,643	1,169	592
		690	59,323	10,000	32,815	1,130	602
	21	10	65,439	10,000	37,066	1,058	847
		25	68,886	10,000	41,655	959	870
		136	60,777	10,000	35,051	1,375	1,060
	23	1.5	29,945	10,000	12,260	1,082	468
		26	54,233	10,000	31,370	1,242	1,004
		45	50,512	10,000	28,488	1,474	905
		182	46,486	10,000	24,155	1,169	483
	24	1	73,158	10,000	30,712	1,091	1,050
		30	55,368	10,000	23,587	1,111	826
		100	48,354	10,000	29,903	1,449	926
		210	66,439	10,000	37,036	1,182	913
	30	27	70,397	10,000	42,877	899	853
		251	22,755	10,000	13,662	1,313	311
	40	1.5	67,634	10,000	25,398	1,183	978
		20	48,904	10,000	19,510	1,252	855
		35	37,672	10,000	22,269	1,201	827
		150	74,555	10,000	41,027	1,421	1,264
		308	51,515	10,000	21,627	1,259	658
	51	1.8	75,111	10,000	31,898	1,032	854
		30	46,130	10,000	27,208	1,203	758
		225	43,482	10,000	24,960	1,313	704
		425	47,272	10,000	27,621	1,260	594
R/V Falkor Cruise 003b July 2012	8	3	28,884	10,000	13,075	738	339
		10.4	37,363	10,000	19,737	804	531
		101	59,413	10,000	29,059	1,182	912
		201	67,232	10,000	33,307	1,013	778
		751	36,029	10,000	16,651	1,013	383
		2690	40,066	10,000	18,233	1,176	455
	10	10.3	36,486	10,000	14,943	947	504
		29	30,200	10,000	16,375	1,071	482
		299	41,661	10,000	22,587	1,038	551
		1046	43,508	10,000	20,630	1,019	468
R/V Knorr Cruise 223 October - December 2014	15	81	54,086	10,000	16,463	497	512
		771	52,444	10,000	16,215	397	254
		3000	37,618	10,000	10,143	376	207
		5505	55,920	10,000	16,146	406	290
