# Supplementary material for: Patterns of Relative Bacterial Richness and Community Composition in Seawater and Marine Sediment Are Robust for Both Operational Taxonomic Units and Amplicon Sequence Variants
Source: Front Microbiol. 2022 Feb 7;13:796758. doi: 10.3389/fmicb.2022.796758 (PMC8859096; doi:10.3389/fmicb.2022.796758)
Supplement: Supplementary file 4 [file Data_Sheet_4.pdf]

Cruise	Site	Sample Depth (mbsf)	Sequences: Raw	Final: OTUs	Final: ASVs	Output: # of OTUs	Output: # of ASVs
R/V Knorr Cruise 223 October - December 2014	15	0.13	53,588	10,000	24,033	925	641
		0.26	45,546	10,000	19,861	903	599
		0.52	41,087	10,000	16,937	732	379
		0.65	43,490	10,000	18,794	687	471
		0.2	42,529	10,000	19,835	878	487
		0.9	42,816	10,000	20,992	871	542
		1.7	48,822	10,000	22,220	561	337
		3.2	38,347	10,000	20,170	708	335
		4.62	54,807	10,000	19,223	385	230
		2.7	67,343	10,000	31,363	598	391
		3.5	56,973	10,000	28,965	613	234
		5.7	54,396	10,000	20,037	628	312
		8	73,270	10,000	40,300	590	226
		9.5	71,647	10,000	33,138	553	307
		11	100,035	10,000	49,824	758	343
		12.5	57,013	10,000	31,228	522	253
		14	83,760	10,000	44,025	630	239
		15.5	67,594	10,000	36,331	654	341
		18.5	24,720	10,000	10,189	253	138
		24.5	68,330	10,000	36,368	462	179
		26	65,221	10,000	33,337	615	289
	16	0.13	57,425	10,000	27,886	835	733
		0.26	61,628	10,000	23,635	721	445
		0.39	54,669	10,000	20,099	482	219
		0.2	54,662	10,000	23,513	385	172
		1.7	49,214	10,000	24,272	833	409
		2.4	44,964	10,000	24,161	675	455
		3.9	45,311	10,000	22,988	829	417
		4.77	69,453	10,000	30,081	818	514
		0.2	43,663	10,000	20,235	827	483
		3.2	51,198	10,000	24,621	859	556
		4.7	46,180	10,000	22,470	888	506
		6.2	19,041	10,000	9,263	766	198
		6.9	62,989	10,000	28,125	708	474
		10.7	66,412	10,000	30,670	692	454
		13.7	57,992	10,000	30,443	704	395
		15.2	53,992	10,000	25,811	835	443
		18.2	59,971	10,000	29,675	607	343
		21.2	78,260	10,000	39,906	733	484
		24.2	54,523	10,000	30,661	540	263
		27.2	61,550	10,000	28,001	796	492
		31.7	89,379	10,000	41,116	634	412
		34.7	52,729	10,000	25,865	655	374
		39.2	49,063	10,000	25,776	398	148
